# Machine learning can aid in prediction of IDH mutation from H&E-stained histology slides in infiltrating gliomas

**DOI:** 10.1038/s41598-022-26170-6

**Published:** 2022-12-31

**Authors:** Benjamin Liechty, Zhuoran Xu, Zhilu Zhang, Cheyanne Slocum, Cagla D. Bahadir, Mert R. Sabuncu, David J. Pisapia

**Affiliations:** 1 Department of Pathology and Laboratory Medicine, Weill Cornell Medicine, New York, NY USA; 2 School of Electrical and Computer Engineering, Cornell University and Cornell Tech, New York, NY USA; 3 School of Medicine, Weill Cornell Medicine, New York, NY USA; 4 Meinig School of Biomedical Engineering, Cornell University, Ithaca, NY USA; 5 Department of Radiology, Weill Cornell Medicine, New York, NY USA

**Keywords:** Tumour biomarkers, Machine learning

## Abstract

While Machine Learning (ML) models have been increasingly applied to a range of histopathology tasks, there has been little emphasis on characterizing these models and contrasting them with human experts. We present a detailed empirical analysis comparing expert neuropathologists and ML models at predicting IDH mutation status in H&E-stained histology slides of infiltrating gliomas, both independently and synergistically. We find that errors made by neuropathologists and ML models trained using the TCGA dataset are distinct, representing modest agreement between predictions (human-vs.-human κ = 0.656; human-vs.-ML model κ = 0.598). While no ML model surpassed human performance on an independent institutional test dataset (human AUC = 0.901, max ML AUC = 0.881), a hybrid model aggregating human and ML predictions demonstrates predictive performance comparable to the consensus of two expert neuropathologists (hybrid classifier AUC = 0.921 vs. two-neuropathologist consensus AUC = 0.920). We also show that models trained at different levels of magnification exhibit different types of errors, supporting the value of aggregation across spatial scales in the ML approach. Finally, we present a detailed interpretation of our multi-scale ML ensemble model which reveals that predictions are driven by human-identifiable features at the patch-level.

## Introduction

With the advancement of computer processing power and the demonstrated utility of deep learning approaches across multiple data-rich domains, the adoption of machine learning to medical diagnostics is anticipated to have a transformative effect on patient care. Already, methylation-based machine learning (ML) approaches to the classification of tumors of the central nervous system (CNS) have demonstrated performance that can exceed traditional histology-based diagnosis^1^, and has allowed for the identification of novel entities^2^ and molecular subtypes within established classification systems^2–4^. Molecularly-defined entities continue to emerge, many demonstrating overlapping histology with other established tumor classes^5^. However, routine histopathologic examination remains the mainstay of oncologic diagnosis due to its low cost, ubiquity, limited availability of molecular testing, and established robustness—particularly when performed by experienced subspecialty expert histopathologists. Even in healthcare centers with access to advanced molecular assays, the availability of subspecialty experts needed to perform organ-specific histopathologic examination and integrate molecular results into the overall diagnostic picture may be lacking. Developing robust machine learning models that leverage the immense, data-rich trove of existing and prospective histology slides via digitally scanned whole slide images (WSI) and that reproduce or augment subspecialist histopathology expertise can (1) help general pathologists render accurate subspecialty diagnoses, (2) serve as a check on human sources of error by acting as a highly reproducible and fatigue-free assistant, (3) help prioritize the highest yield assays for a given specimen, reducing costs and tissue expenditure, and (4) reveal discordant biases between ML models and human pathologists, which when approached synergistically could increase the detection of clinically pertinent biomarkers more reliably than either in isolation. Moreover, interrogating and understanding the features that drive ML classification could reveal avenues for improvement in human expert assessments.

Infiltrating gliomas are the most common primary tumors of the CNS in adults^6,7^. Despite significant advances in the understanding of their biology, they are considered incurable by current standards of care, including surgical gross total resection, radiotherapy, and chemotherapy^8^. Historically, infiltrating gliomas were classified into the broad categories of astrocytoma and oligodendroglioma on cytomorphological grounds, and assigned histologic grades based on particular features including mitotic activity, necrosis, and microvascular proliferation. The term ‘glioblastoma’ (GBM) was synonymous with the highest-grade variant of infiltrating astrocytoma (IV of IV) and such tumors carry a poor prognosis with an average survival less than 2 years^9^. With the discovery of isocitrate dehydrogenase (IDH) mutation as a key driver of gliomagenesis in 25–30% of infiltrating gliomas and its correlation with a favorable prognosis, recent consensus guidelines regard IDH-mutant (IDHmut) tumors as biologically distinct entities from IDH-wildtype (IDHwt) tumors, and indeed the term ‘glioblastoma’ is now only applied to IDHwt infiltrating astrocytomas with high-grade histological/molecular features^1,10–12^. While IDHmut gliomas are enriched for tumors with lower-grade histomorphology, there is no known definitive histologic standard for determining IDH status from histomorphology alone, and immunohistochemical or molecular methods remain the unequivocal gold-standard for such a determination; however, histomorphologic correlates of molecular alterations are well-recognized in many tumor types, including infiltrating gliomas. As noted by the WHO, certain histologic features have a stronger association with IDHmut status, including gemistocytic and oligodendroglial-like cytomorphology, while higher grade features such as palisading necrosis and microvascular proliferation are enriched in IDHwt tumors; however these features lack sensitivity and specificity^10,13,14^. Our experience suggests that subspecialty neuropathologists who review a high volume of infiltrating gliomas can predict the presence of IDH mutation from routine H&E stains with a relatively high degree of accuracy. Therefore, we believed that histological prediction of IDH-status represented an ideal prototype for the more general task of designing computer vision models to interrogate whole-slide images (WSI) to predict clinically relevant tumor biomarkers.

Convolutional neural networks (CNNs) are one of the most popular ML architecture choices for a wide-ranging set of computer vision tasks^15–19^. A challenge in the application of CNNs to WSI processing is that there is a practical limit to the input image size that can be handled (typically less than 1000 × 1000 pixels) by today’s hardware resources, such as GPU compute power and memory. WSI often have in the range of 10^5^ pixels in each dimension, and key diagnostic features are usually seen only in small foci, necessitating tiling of the source image into appropriately sized training patches, and aggregation of patch-level class predictions to generate slide-level predictions. Previous work has shown that CNNs can be used to classify WSI histology data, particularly in epithelial cancers, including the prediction of driver mutations in some cancers^20–29^. Furthermore, integrating CNN predictions from histology with genomic information has been found to predict behavior in infiltrating gliomas better than traditional histologic grading alone^30^.

Prior studies have largely trained ML classifiers on image patches derived at a single level of magnification without aggregating across scales^20–30^. This is in contrast to what pathologists typically do, which is use a range of magnifications in assessing tissue; i.e., pathologists scan slides at low magnification both to identify features better appreciated at low power as well as to identify regions of interest for closer examination at higher power. We therefore hypothesized that the accuracy of our prototypical classification task would be magnification-level dependent, and that ensembling ML models trained at different scales would generate more robust classification. Finally, we hypothesized that neuropathologists and ML models would make different types of errors in classification, and that the aggregate assessment of a hybrid pathologist/ML model would be superior to either human or ML assessment alone.

## Results

### ML models accurately predict IDH mutation status

**Figure 1:**
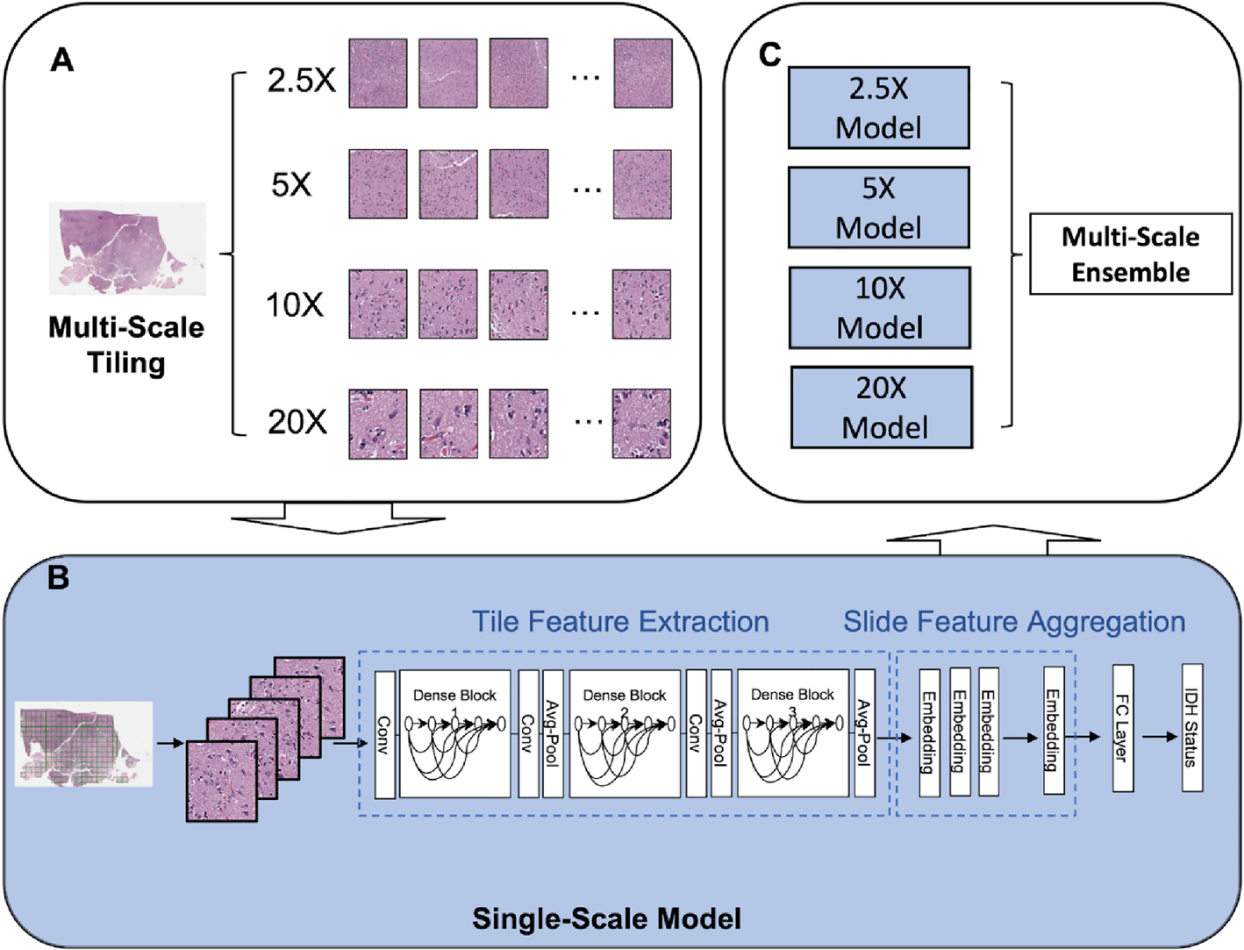
A schematic for the end-to-end process of model training and deployment. WSI are tiled into patches of 256 × 256 size at 2.5×, 5×, 10×, and 20× magnification factors (1**A**). In each training iteration (mini-batch), 200 randomly selected and augmented patches from a single magnification of a single WSI were passed to single-scale Densenet121 classifiers, initialized with imageNet pre-trained weights. Feature embedding vectors from each patch were then aggregated using naïve averaging, and the resulting vector was then passed to a final fully connected (linear) classifier (1**B**). Following training, the predictions three versions of each single-scale model trained with different random seeds were averaged to produce a single-scale ensemble, and the predictions from each single-scale ensemble were averaged to produce the multiscale ensemble (MSE) predictions (1**C**).

WSI images obtained from the publicly available TCGA database were used for training, including 801 (601 IDHwt and 200 IDHmut) slides (Table 1). These were split into training, validation, and test sets. As an external validation set, WSI from our institution (Weill Cornell Medicine) were used, comprising 174 (87 IDHwt and 87 IDHmt) slides. WSI were tiled into 256 × 256 pixel patches over multiple down-sampled levels corresponding to 2.5×, 5×, 10×, and 20× magnification (Fig. 1A; see “Materials and methods” section). Single-scale models were trained using the DenseNet-121 CNN architecture^31^ and patch-level embeddings were aggregated into slide-level embeddings via average pooling, which were then used to generate slide-level IDH mutation probabilities at output. 200 patches from each WSI were randomly selected and passed to the network during each training step (Fig. 1B). A multi-scale ensemble (MSE) was then generated by averaging all the predictions over the single-scale models (Fig. 1C; see “Materials and methods” section for detail).

**Table 1:** Summary of the demographics for the TCGA training, validation, and test datasets and the WCM test datasets.

	Overall	IDH Status		*p* value
		WT	MUT	
**Count (n) Slide (patient)**				
Training	681 (312)	541 (232)	140 (80)	
Validation	60 (29)	30 (13)	30 (16)	
Test	60 (31)	30 (16)	30 (15)	
TCGA Overall	801 (372)	601 (261)	200 (111)	
WCM Test	174 (141)	87 (74)	87 (67)	
**Age (years) Mean (standard deviation)**				
Training	52.5 (16.4)	58.0 (13.1)	36.5 (14.6)	0.131†
Validation	41.5 (19.7)	59.9 (12.1)	26.5 (8.56)	
Test	47.5 (21.0)	62.1 (15.7)	32.0 (13.4)	
TCGA Overall	51.2 (17.3)	58.4 (13.2)	34.5 (14.1)	< 0.0001
WCM Test	52.4 (16.6)	62.7 (12.8)	41.1 (12.5)	< 0.0001
**Female n (%)**				
Training	115 (36.9)	84 (36.2)	31 (38.8)	0.821†
Validation	13 (44.8)	8 (61.5)	5 (31.3)	
Test	13 (41.9)	6 (37.5)	7 (46.7)	
TCGA Overall	141 (37.9)	98 (37.5)	43 (38.7)	0.921
WCM Test	63 (44.7)	38 (51.4)	25 (37.3)	0.132

No significant differences are seen in sex between the IDHmut and IDHwt groups. IDH mutant gliomas show statistically significant enrichment in younger patients, consistent with historic controls.

^†^Average simulation *p*-value: 140 IDH WT slides in the training dataset were randomly sampled and one-way Anova was then conducted. Simulations were repeated 1000 times.

**Figure 2:**
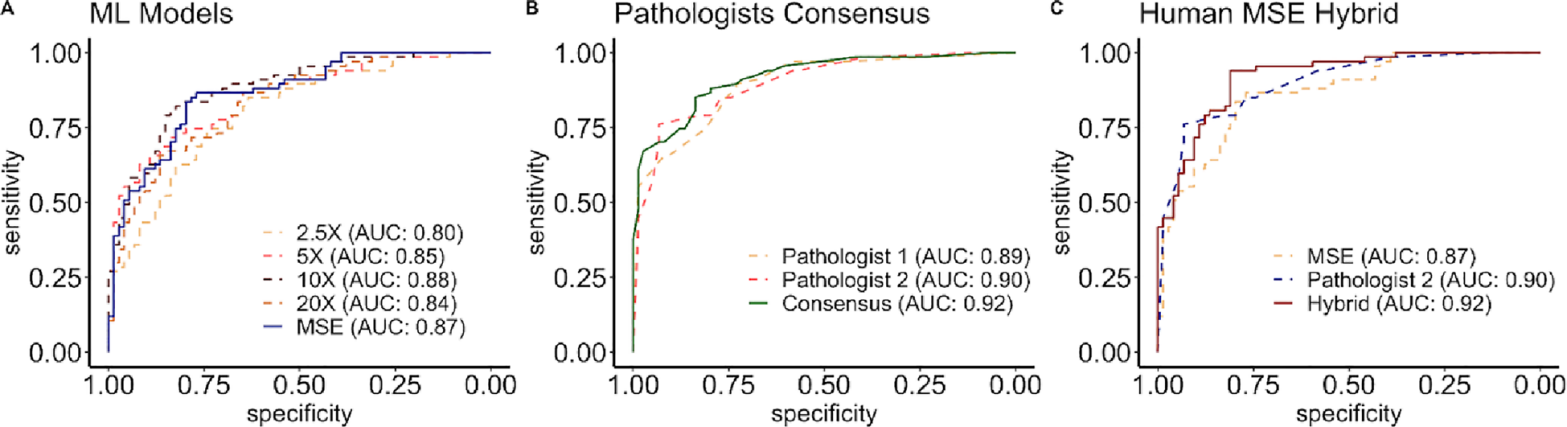
ROC curves for the ML classifiers, pathologists, and hybrid models on the WCM test data. (**A**) compares the model performance of the single-scale ensembles and the multi-scale ensemble. (MSE). The performance of the semiquantitative predictions of two expert neuropathologists and the two-pathologist averaged consensus are compared in **B**). (**C**) compares the predictions of the top-performing neuropathologist with the MSE, and the hybrid model generated by naïve averaging of pathologist and MSE predictions.

Receiver operating characteristic (ROC) curves were generated for patient-level predictions of IDH status evaluated on the WCM test dataset using (1) single-scale models, (2) multiscale ensemble (MSE) ML model, (3) expert neuropathologist, and (4) hybrid neuropathologist-MSE scores. Single-scale models showed differential accuracy, with the peak at intermediate levels of magnification (Fig. 2A) (10× classifier AUC = 0.881, 95% confidence interval = 0.88–0.883), with diminished AUCs seen in models using the lowest (2.5×) and highest (20×) levels of magnification. No ML model demonstrated a superior AUC compared to neuropathologists (Fig. 2B), and consensus averaging of the two neuropathologists’ semiquantitative predictions demonstrated a higher AUC than each neuropathologist individually. Averaging the top performing neuropathologist’s semiquantitative predictions with the MSE prediction scores to generate a human-ML hybrid classifier (Fig. 2C) shows a higher AUC than either the ML classifier or the pathologist alone, and demonstrates performance similar to that of the two-neuropathologist consensus (hybrid classifier AUC = 0.921, 95% confidence interval = 0.920–0.923 vs. neuropathologist consensus AUC = 0.92, 95% confidence interval = 0.918–0.921). Averaging of two-neuropathologist consensus with the ML model provides an incremental increase in prediction accuracy (AUC = 0.928, 95% confidence interval 0.927–0.929). The patient and slide level sensitivity, specificity, and AUC for the individual neuropathologists, two-neuropathologist consensus, the MSE classifier, pathologist-MSE hybrids, and the two-neuropathologist consensus-MSE hybrid, evaluated using the WCM test dataset is summarized in Supplemental Table 1. A full summary of the slide-level and patient-level performance for the single-scale and multi-scale classifiers using the TCGA validation, TCGA test, and WCM test sets is also shown in Supplemental Table 2.

### Single-scale ML models make distinct errors relative to each other and to humans

**Figure 3:**
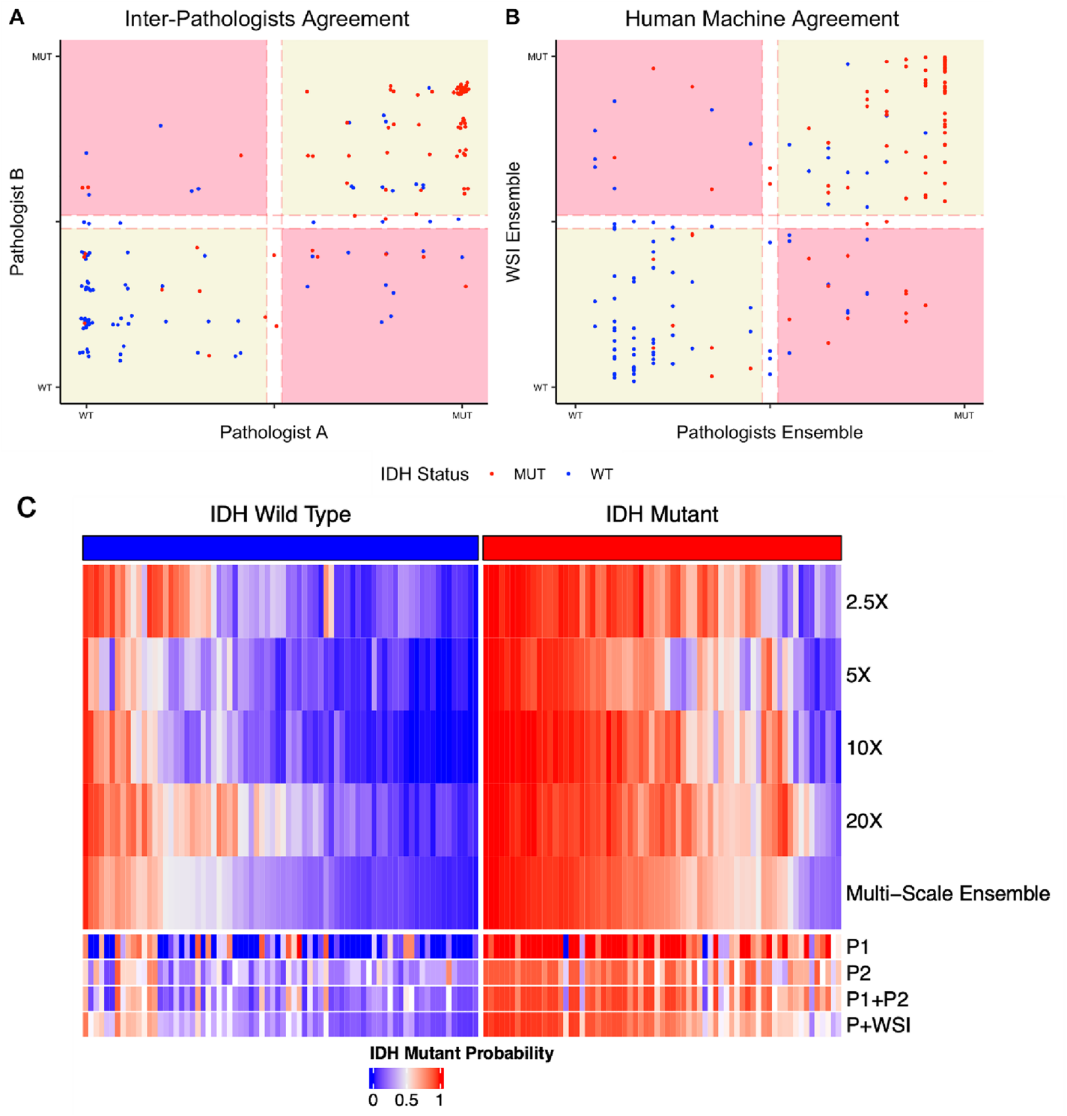
Patient-level predictions in the WCM test data, for the pathologists and ML models. Panel (**A**) compares the semiquantitative prediction scores of the two neuropathologists (κ = 0.656, R = 0.767). Panel (**B**) compares the two-neuropathologist consensus predictions to the multiscale classifier. (κ = 0.598, R = 0.674). Panel (**C**) shows all patient-level predictions using the single-scale models, multiscale ensemble, individual pathologists (P1, P2), two-pathologist consensus (P1 + P2), and the hybrid classifier (P + WSIP1 + MSE). Software utilized the ComplexHeatmap R package (https://doi.org/10.1002/imt2.43) and R version 4.0.3 (2020-10-10).

Comparisons of patient-level predictions of the pathologists and classifiers using the WCM data are shown in Fig. 3. Figure 3A shows a scatter plot comparing the semiquantitative prediction scores of the two pathologists. Concordant predictions are found in the yellow quadrants, while discordant predictions appear in the pink quadrants. High densities of accurate predictions are located at the extremes of the concordant regions, while inaccurate predictions are enriched in regions of lower certainty. The Pearson coefficient R for the semiquantitative predictions of the pathologists is 0.767, while the Cohen’s kappa for the binary predictions of the pathologists is 0.656. Figure 3B shows a scatter plot of the pathologist consensus score (averaged semiquantitative predictions of the pathologists) compared to the MSE predictions. The correlation between MSE and pathologist consensus is less than between the two pathologists (Pearson coefficient R = 0.674), and correspondingly there is a lower degree of concordance between the binary classifications (Cohen’s kappa = 0.598). Among discordant cases, there is a slight enrichment of IDHmut cases that are accurately predicted by the pathologists and missed by the MSE, while there is slight enrichment of IDHwt cases accurately predicted by the MSE and missed by the pathologists. Figure 3D shows patient-level IDH prediction scores from the single-scale and multi-scale ensemble classifiers, pathologists, and hybrid predictions, highlighting the orthogonal nature of errors made at individual levels of magnification. A heatmap of the slide-level predictions from each classifier is shown in Supplemental Fig. 1. A matrix comparing the kappa scores of all ML classifiers, pathologists, and the hybrid classifier are shown in Supplemental Fig. 2.

### Patch-level predictions reveal features that drive accurate and inaccurate predictions

**Figure 4:**
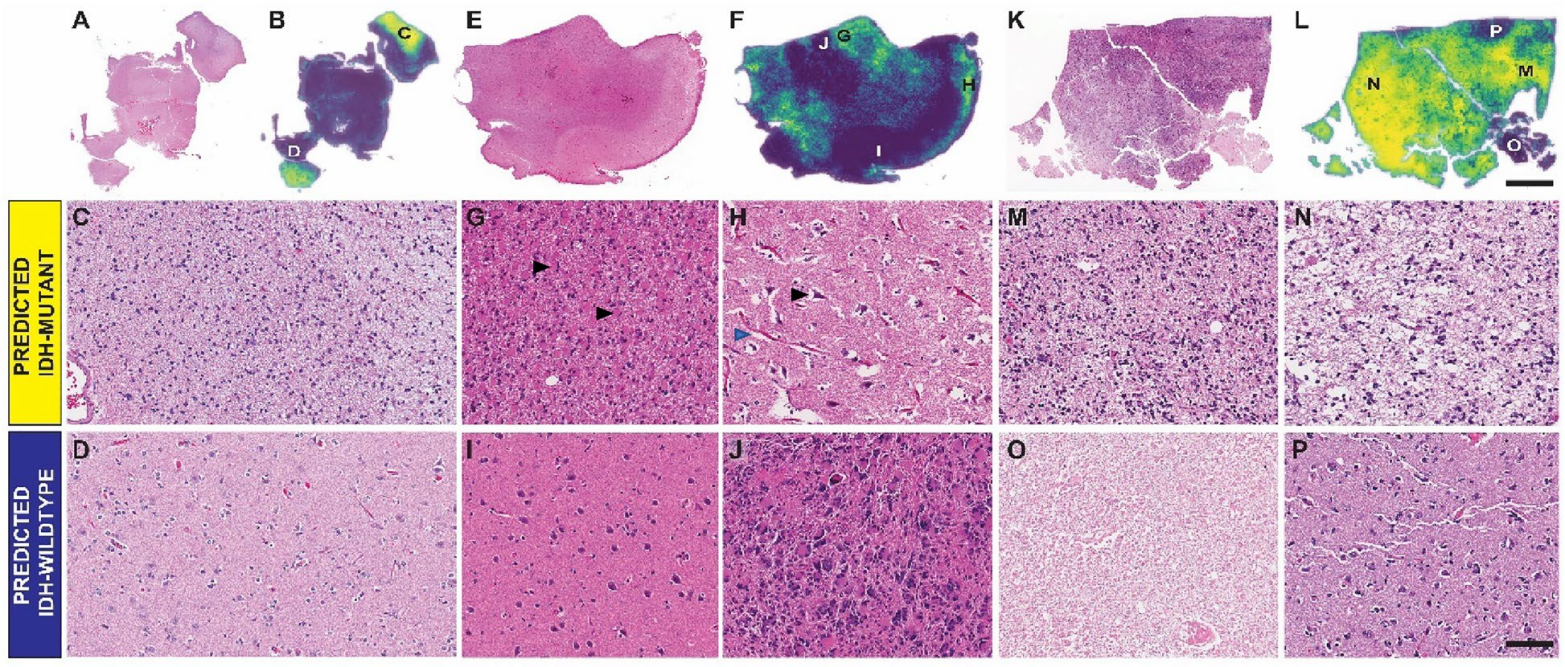
Shows examples of the sliding windows visualizations, with representative patches from regions from 3 example cases that provide insight into features recognized by the classifier. 4(**A**) show a low power H&E image of a slide that was accurately predicted as IDHmut by the neuropathologists, but was incorrectly classified by the MSE. 4(**B**) shows a heatmap of average pixel-level IDH mutation status predictions. Selected patches from image 4(**A**) demonstrate higher IDHmut predictions in regions of solid tumor (4**C**), with higher IDHwt predictions in regions of minimally involved brain parenchyma (4**D**). 4(**E**) and 4(**F**) show an example of a slide from an IDHmut case, which was misclassified by both the neuropathologists and the ML classifier. Regions from this slide containing tumor with monomorphic gemistocytic cytomophology (4**G**; arrows = examples of gemistocytic cells) and regions of minimally involved brain parenchyma with perineuronal (black arrow) and perivascular (blue arrow) white space artifact (4**H**) were associated with a higher prediction for IDHmut, while areas of minimally involved brain parenchyma without significant whitespace artifact (4**I**) and regions with more bizarre cytology (4**J**) were associated with a higher prediction of IDHwt status. Figures 4(**K**) and 4(**L**) show a slide from an IDHmut glioma which was accurately predicted by the ML classifier, but inaccurately predicted by the neuropathologists. Areas of mildly cellular tumor, both with and without whitespace artifact [4(**M**) and 4(**N)** respectively] were associated with higher IDHmut predictions, while regions of necrosis (4**O**) and regions of minimally involved brain parenchyma (4**P**) were associated with higher IDHwt predictions.

To gain insight into (1) the decision-related morphological features of the ML models and (2) the types of errors made by both the classifiers and pathologists, sliding patch-level IDH predictions were generated for selected slides using the MSE, three of which will be examined in further detail here (Fig. 4). In the first informative case (Fig. 4A–D), neuropathologists were correct in predicting IDH mutation, but the case was inaccurately predicted by the MSE to be IDHwt at the slide-level. Regions shown in yellow (Fig. 4C) were predicted by the MSE as consistent with IDH mutation, and were also recognized by the neuropathologists as harboring relatively hypercellular infiltrating tumor that was likely IDH-mutant. Regions encoded in blue (Fig. 4D) drove the overall slide-level misclassification of MSE. These regions were enriched in brain parenchyma without definitive infiltration by tumor cells (as determined by human examination) and were disregarded as non-contributory to the classification task by the neuropathologists. Although the classifier was correct in determining that these areas were not enriched for IDH-mutated tumor, the binary classification task of determining the slide’s overall IDH status was evidently hampered by the large presence of uninvolved brain.

In a second case (Fig. 4E–J), that of an IDHmut glioma that was inaccurately classified by both the neuropathologists and the MSE, many regions harbored a relatively monomorphic gemistocytic cytomorphology (Fig. 4G). These regions were accurately interpreted by the classifier as consistent with IDHmut status, and in retrospect also likely would have been favored to represent IDH-mutated tumor to the neuropathologists if presented in isolation. However, one region of marked nuclear pleomorphism (4J) was interpreted by both the classifier and the neuropathologists as representing IDHwt tumor, driving the misclassification. Human-determined ‘uninformative regions’ again drove inaccurate MSE classification of particular areas: regions of uninvolved brain but with increased white-space around individual neurons and vascular channels due to tissue processing artifacts and/or edema were predicted as IDHmut (4H) by the MSE, while regions of relatively uninvolved brain and without significant intraparenchymal white-space (4G) were again erroneously predicted as IDHwt as before.

The final example (Fig. 4K–P) illustrates an IDHmut glioma inaccurately predicted by the neuropathologists as IDHwt, but correctly predicted by the MSE. In this case, solid regions of tumor (4M and 4N) were accurately predicted by the ML classifier as areas with (IDH-mutated) tumor. A large area of necrosis was present in this slide (4O), which drove inaccurate prediction of IDHwt by both neuropathologists, and this area in isolation was also classified as IDHwt by the MSE. Once again, the MSE interpreted regions of minimally involved normal brain (4P) as IDHwt. Additional heatmap examples are provided in Supplemental Fig. 3. Heatmaps demonstrating differences in pixel-level predictions at 2.5× versus 20× are provided in Supplemental Figs. 4,5, and highlight scale-dependent differences in IDH-confidence in different areas of the slides.

**Figure 5:**
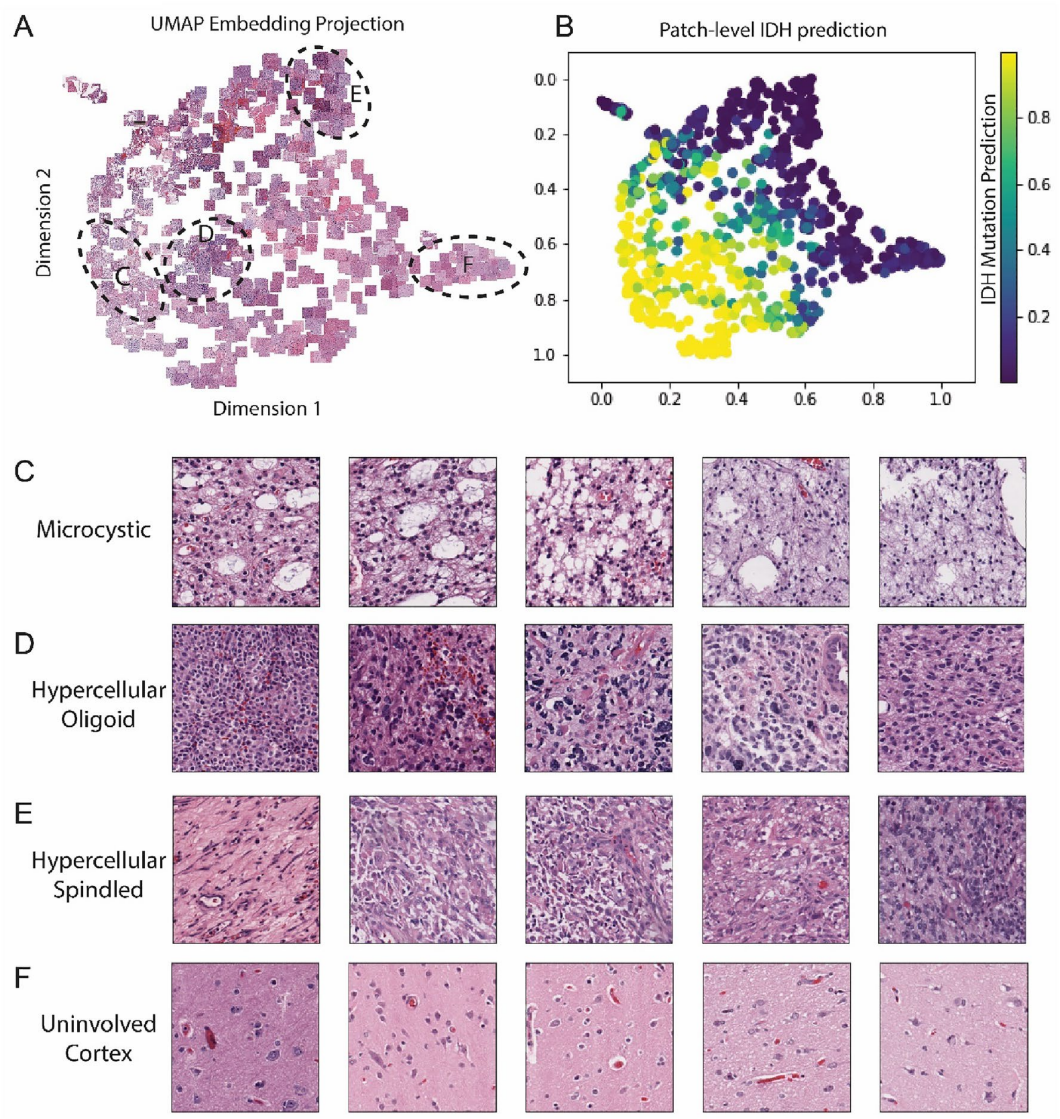
UMAP coordinates of the feature embedding vector activations from patches passed through the 10 × classifier. **A**) shows some example tiles in 2D UMAP coordinates. (**B**) shows the patch-level IDH status prediction scores as predicted by the 10 × classifier. Tiles from region (**C**) demonstrate microcystic architecture. Tiles from region (**D**) demonstrate hypercellular regions of infiltrating tumor, with round cytology, enriched for tumors with oligodendroglial morphology. Tiles from region (**E**) demonstrate hypercellular regions of tumor with a greater degree of nuclear spindling/elongation and nuclear pleomorphism. Tiles from region (**F**) demonstrate brain parenchyma without significant infiltration by tumor cells.

### Patch-level embedding vectors reflect diagnostically relevant human-identifiable features

To gain further insight into the histological features encoded by our trained ML models, 5 random patches were selected from each slide in the WCM dataset and uniform manifold approximation and projection (UMAP) was performed on the patch-level embedding vectors from the best performing 10x-scale classifier (Fig. 5A–B). Review of histological features in clustered patches revealed consistent patterns across patches obtained from distinct slides. Emergent human-identifiable features included: (1) microcystic architecture (Fig. 5C), which is correlated with IDHmut status, (2) hypercellular regions of tumor with round, monomorphic nuclei, reminiscent of oligodendrocytes, which were appropriately enriched for IDHmut tumors (Fig. 5D), (3) hypercellular tumor areas with spindled nuclei and greater pleomorphism, enriched for IDHwt tumors (Fig. 5E), and (4) brain parenchyma without significant human-detectable involvement by tumor (by H&E), that were predicted by the classifier as harboring IDH mutation irrespective of the ground truth slide-level class (Fig. 5F). Other features captured by the embedding vectors include patches with a significant amount of whitespace (Fig. 5A, top-right) and regions with abundant hemorrhage or necrosis (Fig. 5A, top center). The ground-truth IDH-status and integrated molecular diagnosis of the UMAP coordinates are shown in Supplemental Fig. 6. A high-resolution version of Fig. 5A is available upon request.

## Discussion

Just as the molecular classification of neoplastic disease and its impact on patient care have emerged rapidly in the last decade, ML techniques and computational resources continue to progress. An open question in medical diagnostics is whether existing data-rich resources such as WSIs, effectively encodes untapped information that could be leveraged to guide patient management while minimizing the use of more advanced but less accessible modalities. We selected the task of predicting IDH mutation in infiltrating gliomas as a prototypical problem within this space, using CNN models with H&E-based histological information as the sole input. Moreover, we compared the performance of this task over multiple magnification scales. As a reference point, we compared the performance of the CNN models, trained on the order of hours, with those of subspecialty-trained expert neuropathologists, trained on the order of years to decades. While the models demonstrate very high accuracy on the TCGA dataset, a significant drop in performance is seen when applying the same models to the WCM dataset. We believe this is a result of recurrent batch effects in tissue fixation, processing, and staining between different laboratories, and that the overperformance on TCGA data likely represents an element of overfitting on batch effects from a relatively small number of centers, and the performance on the WCM dataset is likely more representative of the performance that would be seen real-world deployment on samples from laboratories to which the models are naïve.

Comparison of ML model predictions to expert pathologists shows that while similar degrees of accuracy are obtained on the classification task, the types of errors made were distinct, combining pathologist predictions and ML predictions results in greater classification robustness than either alone. Manual interrogation of patch-level predictions demonstrates several confounders exploited by the ML models which, interestingly, were found to be reproducible at all levels of magnification. The most striking source of errors in our models were regions of human-interpreted low informativity within the underlying tissue. Specifically, regions of brain without definitive tumor cells were often classified as IDHwt, while regions with increased white-space secondary to vacuolation, edema, and/or tissue artifacts were often classified as IDHmut. While areas lacking tumor are indeed IDHwt per se given the putative absence of tumor cells, the task was built around slide-level classification of de facto tumors. Our interpretation, therefore, is that classifying these regions as IDHwt on-average drove the classifier to a higher degree of accuracy overall, despite patch-level ‘uninformativeness’ as determined by human observers. One approach to address the confounding effects of such regions is to explicitly annotate and train toward a third-class label, that of “non-neoplastic brain” from autopsy and epilepsy cases. Surprisingly, in the set of sliding window heatmaps analyzed, the models were not clearly driven by features that pathologists often used to predict IDH-class due to their enrichment in IDHwt tumors, such as well-formed palisading necrosis and microvascular proliferation.

The presence of human-identifiable features as seen in the UMAP projections demonstrates that the CNNs can recognize some of the features used by humans in the classification of gliomas. We found that patches demonstrating microcystic architecture or oligodendroglial cytomorphology were enriched for IDHmt classification while patches with increased spindled cells and pleomorphism were enriched for IDHwt classification. Histomorphologic correlates of certain driver alterations have been previously identified, such as giant-cell morphology in IDHwt glioblastomas harboring *TP53* mutation, and epithelioid morphology in high grade gliomas harboring *BRAF* mutations; however, given the heterogeneity of infiltrating gliomas, and particularly in IDHwt astrocytomas/glioblastomas, these morphologic correlates as assessed by human pathologists have relatively poor predictive utility^32–37^. The UMAP also clearly illustrated that regions of human-interpreted low informational value relative to the task were enriched for particular classes, such as normal appearing brain being enriched for IDHwt class. Again, the identification of recurrent confounders across these models suggests that strategies to devalue or exclude uninformative patches could further improve classification accuracy, and expanding the number of available classes to include non-neoplastic samples, as alluded to above, may improve ML performance. In addition, we believe that given a sufficiently large dataset of histologic data paired with RNA transcriptome and DNA methylation profiling, histomorphologic correlates may be identified, however further studies will be necessary to assess for this.

Methods to aggregate patch-level predictions into slide-level classifications are a widely studied problem in the multiple-instance learning literature. Attention mechanisms that increase the weight of highly informative patches on the final classification prediction have been found to be useful in other cancer types^22,38^. However, the differing biological characteristics of tumor types that are reflected in histology (for example that infiltrating gliomas typically have an ill-defined border with respect to the surrounding non-neoplastic tissue, a feature that differs significantly from that of epithelial cancers) are likely to impact the efficacy of any particular ML algorithm, and the strategies employed are unlikely to be universally applicable to models trained for all diagnostic tasks. In our experiments conducted with this dataset, we also tested attention pooling mechanisms to aggregate patch-level embeddings into slide-level embeddings using the method described by Ilse et al.^38^ (https://arxiv.org/pdf/1802.04712.pdf), where weights for each patch embedding are learnable; however, this attention mechanism did not provide a significant improvement on classification performance relative to naïve averaging of embedding weights (data not shown). That said, as the number of potential target outputs of the model increases, attention mechanisms may help boost performance, but future studies using a broader variety of target classes are necessary to better assess this.

Our results demonstrate that the level of magnification used for input images impacts the ML model accuracy, with the greatest levels of accuracy achieved at intermediate levels of magnification (corresponding to 10 × objective in our study). One interpretation of this finding is that while lower levels of magnification provide a larger field of view with a greater degree of overall tissue sampling and increased architectural information, higher levels of magnification provide increased cytologic detail yet with a smaller field of view. Intermediate levels of magnification may represent a “sweet-spot” capturing both low-power and high-power information. Of practical importance, some errors made by models using different levels of magnification were found to be orthogonal, and to the errors made by human observers, providing a rationale for multi-scale ML models and hybrid ML-human approaches. We also believe that designing ML models to explicitly recapitulate the human methodology of examining the tissue at lower power, and then selecting regions of interest to interrogate at higher power could result in more robust model predictions, while also using less computational resources than interrogating an entire image at high power. However, future studies will be necessary to confirm this.

While routine H&E staining is not used to make determinations of mutational status, its global availability, low cost, and diagnostic richness have established it as a mainstay of surgical pathology for over a century. Immunohistochemistry for the most common pathogenic IDH mutation (IDH1 R132H) detects 85–90% of IDH mutant gliomas, with the remainder requiring DNA sequencing to identify. Of note, in our cohort, all slides harboring non-R132H IDH-mutations were correctly classified by our models (n = 6, IDH1 R132C = 4, IDH2 R172K = 2). This work suggests that computer vision-based approaches may assist in subclassification of tumors for which gold-standard molecular diagnostics are not universally available and in selecting assays for additional testing.

Studies evaluating at the ability of ML models to predict IDH mutation status have been previously published. Jiang et al.^39^ found that WSI could be used to predict IDH mutation status and survival in gliomas with grade 2 and 3 histology. Liu et al*.* found that the inclusion of a generative adversarial network (GAN) to augment training data and including patient age as a model input could both improve model accuracy. Both these studies used TCGA glioma cohorts for model training. To our knowledge, our work is the first to evaluate aggregate expert human predictions with model predictions, and to compare the features learned by ML models with those that have been identified as predictive by human pathologists, and to compare the predictions of ML models at multiple levels of magnification. Further studies will benefit from larger image slide datasets including greater variability of laboratory-specific staining protocols. In addition to training models to detect particular clinically-relevant molecular alterations, of interest will be to train models directly toward patient outcomes in an effort to disclose previously unappreciated histological features of clinical and prognostic relevance.

This study demonstrates that ML models can achieve near human-level performance at predicting clinically relevant oncologic biomarkers of CNS tumors using H&E-based histological information alone, even with a completely external test set, with training times and slide exposure that is minimal compared to that needed to train human subspecialty experts. Moreover, by analyzing single magnification and multi-scale models and interrogating encoded features through heatmap and UMAP visualizations of patch-level predictions, crucial insights of how to iteratively improve the ML models can be obtained. Our study represents a proof-of-principle that ML models hold great promise in approaching and potentially superseding human level performance of biomarker detection via deep learning of widely accessible H&E slides, paving the way to uncovering the full diagnostic and prognostic potential of this ubiquitous data modality.

## Materials and methods

### Human subjects research

This research and experimental protocols were conducted in accordance with Weill Cornell Medicine’s Institutional Review Board requirements under the IRB-approved protocol #1312014589. All patients were initially consented to surgical procedures from which slide image data was obtained as per institutional guidelines. The IRB for this research itself was approved with a waiver of consent given its retrospective nature and given there was no contact with patients, and the research conforms to the ethical requirements and HIPAA compliant protections mandated by the institutional IRB.

### Dataset

In this study, we used datasets from two cohorts of infiltrating gliomas patients obtained from The Cancer Genome Atlas (TCGA)^40^ and Weill-Cornell Medicine (WCM). (1) TCGA: We downloaded H&E-stained WSI along with gender and age information from the TCGA-LGG and TCGA-GBM datasets. Clinical data for the merged TCGA LGG and GBM cohort was downloaded from cbioportal (date of download September 17, 2020). Cases without reported IDH mutation status, or without formalin-fixed paraffin-embedded (FFPE) H&E-stained slides available for download were excluded. From these datasets, we obtained a total of 801 slide images (601 IDHwt and 200 IDHmut) from 372 patients (261 IDHwt and 111 IDHmut) (Table 1). We then split TCGA data into training, validation, and test sets, with all slides from individual patients being sorted to the same subset. To ensure IDH class balance during model evaluation for straightforward interpretation, we randomly sampled 30 IDHwt slides and 30 IDHmut slides each in both the TCGA validation and test sets. All other slides in the TCGA cohort were used for training. (2) WCM: We queried the in-house clinical database at WCM for infiltrating gliomas with available H&E-stained slides, with recorded IDH mutation and 1p19q codeletion status, from 2011 to 2020. From these cases, a balanced dataset of IDHwt and IDHmut gliomas (including both astrocytomas and oligodendrogliomas) were scanned using the Aperio T2 system at 40X. This test dataset comprised 87 slides from 74 patients with IDHwt gliomas, and 87 slides from 67 patients with IDHmut gliomas. The images were reviewed by author CS for quality, and the evaluating authors (BL and DP) were blinded to all information about the cases beyond the scanned H&E slides. The WCM dataset was used as an independent external test set to evaluate ML model robustness and generalizability and to compare the ML models with human IDH prediction performance.

### Image preprocessing

We first tiled all WSI into non-overlapping patches of size 256 by 256 pixels at spatial resolutions corresponding to 2.5×, 5×, 10×, and 20× magnification (Fig. 1A). Pixel values ranging between 40 and 215 in greyscale space were treated as informative tissue, and pixels outside this range were considered uninformative, either as background whitespace (> 215) or folded tissue (< 40). Only patches with over 75% tissue percentage were kept for further training and testing. All patches with significant blurriness or pen marks were excluded by thresholding RGB values obtained heuristically.

### Image augmentation

To increase the model generalizability and reduce potential overfitting, we implemented several image augmentation strategies during training. Since all patches within each batch were from one WSI, color augmentations were performed on slide level for each iteration, i.e., we only used one set of color augmentation parameters each iteration for all patches from each slide. We first transformed RGB patches into HSV color space. Then pixel values were augmented channel-wise as: $I_{c}^{aug} = \alpha_{c} I_{c} + {\upbeta }_{{\text{c}}}$. $I_{c}$ were pixel values in channel $c$. $\alpha_{c}$ and ${\upbeta }_{{\text{c}}}$ were channel specific color augmentation factors. ${\upalpha }_{{\text{c}}}$ and ${\upbeta }_{{\text{c}}}$ were sampled uniformly from $U\left( {1 - \sigma , 1 + \sigma } \right)$ and $U\left( { - \sigma , \sigma } \right)$ respectively for each slide. We set $\sigma$ as 0.05 to control augmentation degree. In addition, each patch had 50% probability of being flipped either vertically or horizontally and equal probability (25% each) of being rotated by 0, 90, 180 or 270 degrees. Distinct augmentation parameters were randomly generated during patch selection for each mini-batch.

### Model training

After the image preprocessing step, each WSI had four sets of patches corresponding to magnifications of 2.5×, 5×, 10×, and 20×. Single-scale models were trained for each scale. We used a pre-trained DenseNet-121 architecture^31^, without the last dense layer, as the feature extractor to generate patch-level embeddings of length 1024. All patch-level embeddings from one slide generated in each iteration were aggregated into slide-level embeddings using average pooling. A randomly initialized fully connected layer with 1024 nodes was then implemented to take the aggregated slide-level features as input and output slide-level IDH mutation probabilities. Due to memory constraints, only 200 patches from one WSI were randomly selected and passed to the network for each training step (Fig. 1B). If there were less than 200 patches for one slide, we used all available patches in that mini-batch. Note the mini-batch consisted of a single WSI. To keep IDH classes balanced during training, we randomly sampled 140 IDHwt slides and used all 140 IDHmut slides in each training epoch. We used Adam as to minimize binary cross-entropy loss^41,42^ with a learning rate of 0.00001, and a maximum of 100 epochs^43^. All network parameters, including the weights of the DenseNet-121 backbone were updated during training. Models from the epoch with the best validation loss were used. Three separate single-scale models were trained using different random initial seeds.

### Model inference

The trained models from the last step can be used for predicting both patch-level and slide-level IDH mutation status. We first averaged the three slide-level probabilistic predictions at a given scale to compute single-scale predictions. A multi-scale ensemble (MSE) was then computed by averaging all four single-scale predictions (Fig. 1C). For patients with multiple slides, patient-level predictions were computed by averaging slide-level predictions. For measures of prediction accuracy, a threshold of 0.5 was used as a cutoff for IDHmut status.

### Pathologist evaluation

The WCM test set was separately evaluated by two neuropathologists (authors BL and DP), blinded to all patient information and ancillary testing beyond the WSI, to compare the model predictions to human observers. For each case, both pathologists were asked to issue a prediction for IDH status in a semiquantitative scale, normalized to a range of 0 and 1 (i.e., 0 for a prediction of IDHwt and 1 for IDHmut, values close to 0.5 for cases with low certainty). The pathologists’ predictions were then averaged to generate a two-pathologist consensus score. The predictions from each pathologist were averaged with the MSE prediction to generate hybrid classifier scores, and the two-pathologist consensus score was averaged with the MSE predictions to generate a two-pathologist consensus-hybrid model.

### Prediction heatmap

Eight cases in the WCM test set that represent all possible IDH status combinations of ground-truth, pathologists’ ensemble, and slide-level MSE predictions, were selected for heatmap visualization. We used a sliding window strategy to generate a MSE prediction heatmap. We set window size as 256 × 256 and step size as 256, 128, 64 and 32 for 20×, 10×, 5×, and 2.5×, respectively. Using this sliding windows process, we passed patches containing greater than 50% tissue pixels through the single-scale models. Pixel-level predictions were computed by averaging model predictions for patches that contained that pixel, excluding patches below the 50% tissue threshold. These heatmaps were then manually examined by pathologists to gain insights into the histologic features impacting predictions. Pixel-level predictions at high and low magnification were compared by subtracting predictions obtained by the 2.5 × model from the predictions from the 20 × model. Software utilized the matlibplot 3.6.2 Python package available at https://matplotlib.org. Source code used for generation of sliding window figures is available at https://github.com/Karenxzr/IDHmut/blob/main/Visualize.py.

### UMAP visualization

We randomly selected five 10× patches from each WSI in the WCM test set for UMAP visualization^44,45^. Patch embeddings extracted by trained convolutional base of the best performing 10× classifier were used as patch representations. We used the Python UMAP package with default hyper-parameters to obtain the UMAP representations for each patch. For visualization purposes, the first two dimensional vectors of UMAP projections were used as coordinates to show the original input patches, ground-truth IDH mutation status, ground-truth integrated molecular diagnosis (oligodendroglioma, IDHmut astrocytoma, IDHwt astrocytoma), patch-level IDH prediction scores, and slide-level IDH prediction scores from the classifier. The patches were then reviewed by the pathologists to determine the presence of human-identifiable features in each clustering, and the association between histomorphology with specific diagnoses.

### Statistical analysis and software

All model trainings and inferences were performed on 4 NVIDIA Titan X GPUs. Image preprocessing, model training and inference were conducted in Python, version 3.7.4. OpenSlide python was used for reading and tiling WSI. Pytorch was used for training neural networks. All statistical analyses were performed in R, version 4.0.3. Slide prediction heatmaps were plotted using the ComplexHeatmap R package^46^. Age differences were evaluated using t-test. Chi-square test was used to test the gender difference between two IDH status groups. Confidence intervals of model performance metrics were evaluated through sample bootstrapping for 1000 times. All statistical tests were two-sided with a significance threshold of *p* < 0.05.

### Significance

We show that combining an expert pathologist’s assessments with ML model predictions can classify IDH mutation status in infiltrating gliomas at a comparable level to two-expert consensus. Our study is a proof of principle for the broader application of ML models in deriving clinically relevant molecular markers based on histopathology alone. We also demonstrate that ML-based histopathology classification accuracy varies with level of magnification, and discordant errors are made across scales. This suggests value in ensembling across levels of magnification.

## Supplementary Information

Supplementary Information 1.

Supplementary Information 2.

## Data Availability

All TCGA histology image data used in this study is publicly available through https://portal.gdc.cancer.gov/repository. De-identified metadata corresponding to the WCM histology image dataset (WSI database) is available upon request. Raw scanned *.svs files corresponding to the WCM histology image dataset may be shared in accordance with institutional guidelines including development of an institution-specific Materials Transfer Agreement and in accordance with appropriate HIPAA-compliant interinstitutional IRB-approved protocols.

## Code availability

All source code and guidelines are publicly available on https://github.com/Karenxzr/IDHmut.
