# Supplementary material for: Machine learning can aid in prediction of IDH mutation from H&E-stained histology slides in infiltrating gliomas
Source: Sci Rep. 2022 Dec 31;12:22623. doi: 10.1038/s41598-022-26170-6 (PMC9805452; doi:10.1038/s41598-022-26170-6)
Supplement: Supplementary file 1 — Supplementary Information 1. [file 41598_2022_26170_MOESM1_ESM.docx]

**Supplemental Figures:**

**Supplemental figure 1:**


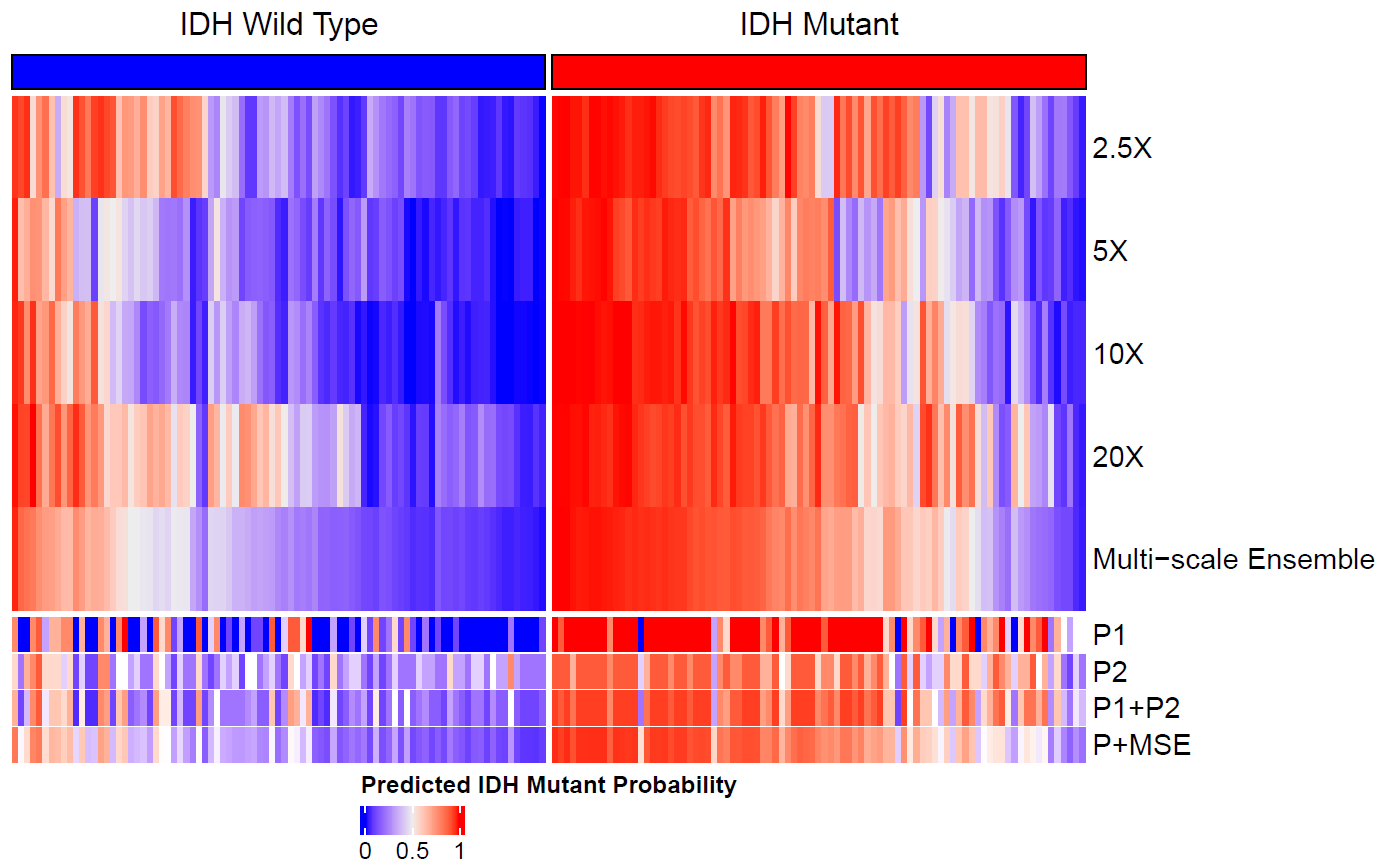


**Supplemental figure 1:** Slide level heatmap of hierarchical clustering comparing prediction scores from all ML classifiers, individual pathologists, two-pathologist consensus, and hybrid predictions, evaluated on the WCM test dataset. Software utilized the ComplexHeatmap R package (https://doi.org/10.1002/imt2.43) and R version 4.0.3 (2020-10-10).


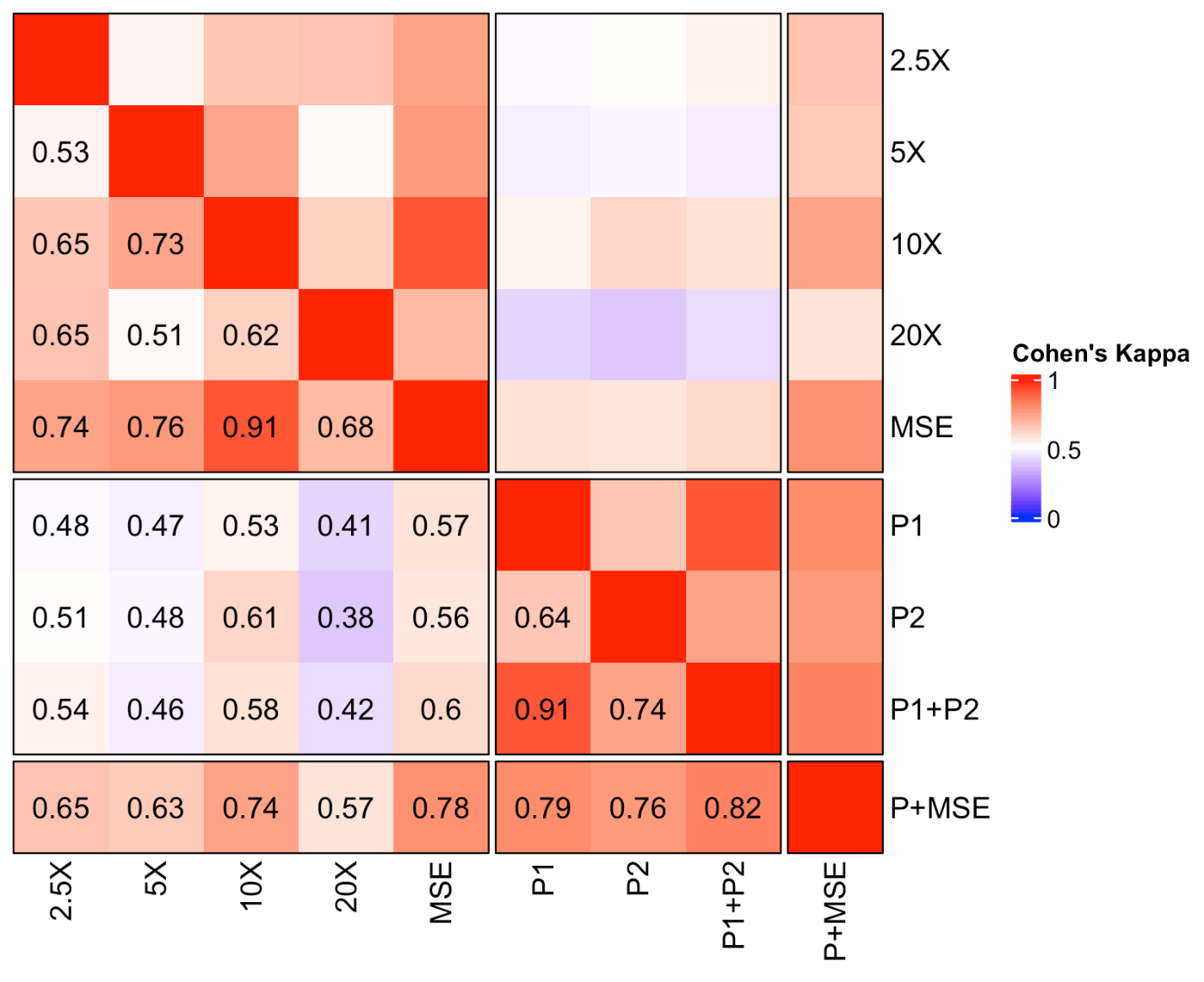


**Supplemental Figure 2:** Cohen’s kappa for binary classifications from all ML models, pathologists, and the hybrid model.


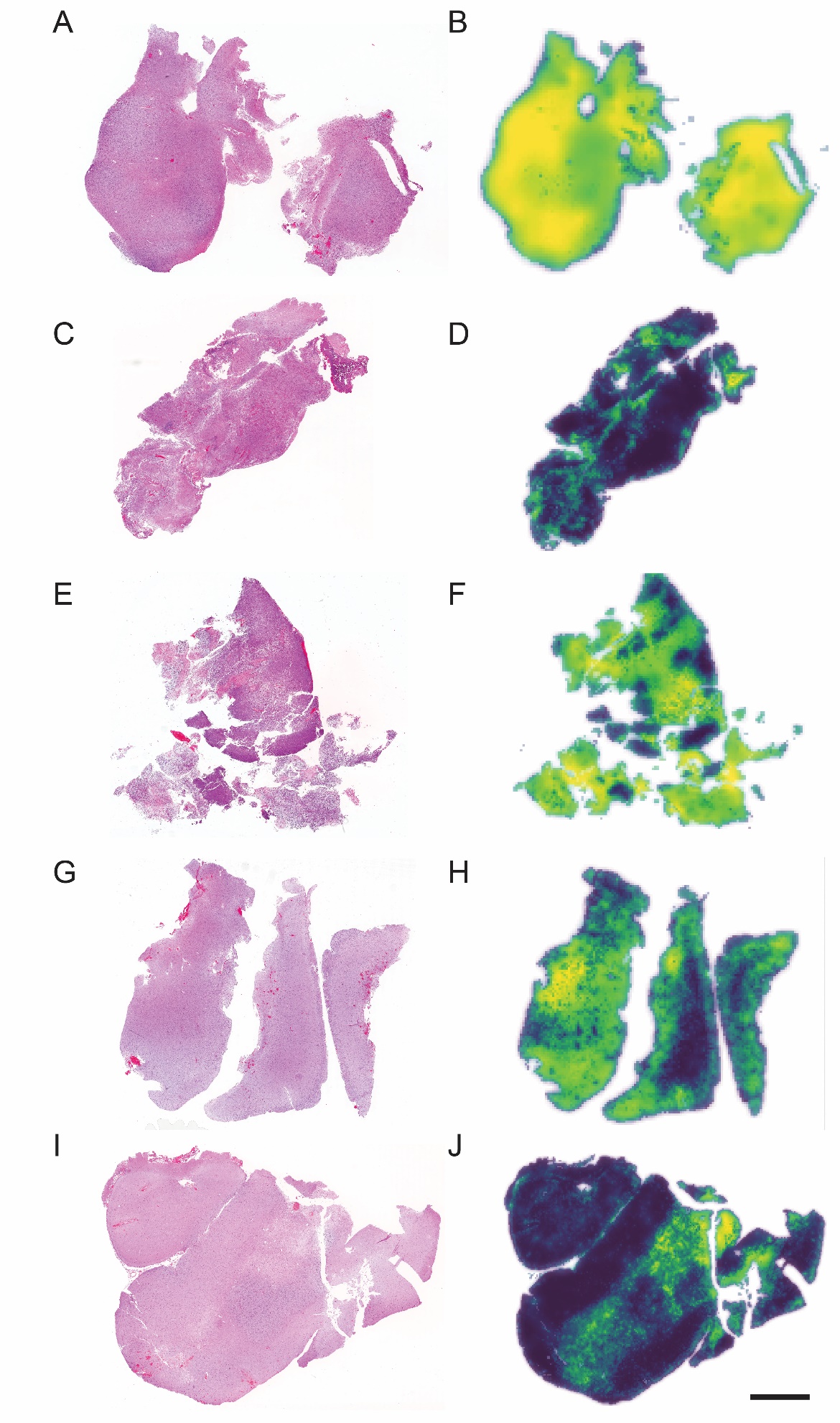


**Supplementary Figure 3.**Additional example sliding window heatmaps of the MSE IDH predictions.
The left panels show whole slide images of the H&E-stained tissue at low power. The right panels show the corresponding MSE prediction maps. Yellow corresponds to MSE predictions of IDH-mutant while blue corresponds to IDH-wildtype. Different combinations of values for slide-level ground-truth, MSE predictions and human ensemble assessments are shown:
A-B: Ground truth = IDHmt; MSE prediction = IDHmt; human ensemble assessment = IDHmt.
C-D: Ground truth = IDHwt; MSE prediction = IDHwt; human ensemble assessment = IDHwt.
E-F: Ground truth = IDHwt; MSE prediction = IDHmt; human ensemble assessment = IDHwt.
G-H: Ground truth = IDHwt; MSE prediction = IDHmt; human ensemble assessment = IDHmt.
I-J: Ground truth = IDHwt; MSE prediction = IDHwt; human ensemble assessment = IDHmt. Software utilized the matlibplot 3.6.2 Python package available at https://matplotlib.org. Source code for sliding window software is available at https://github.com/Karenxzr/IDHmut/blob/main/Visualize.py.


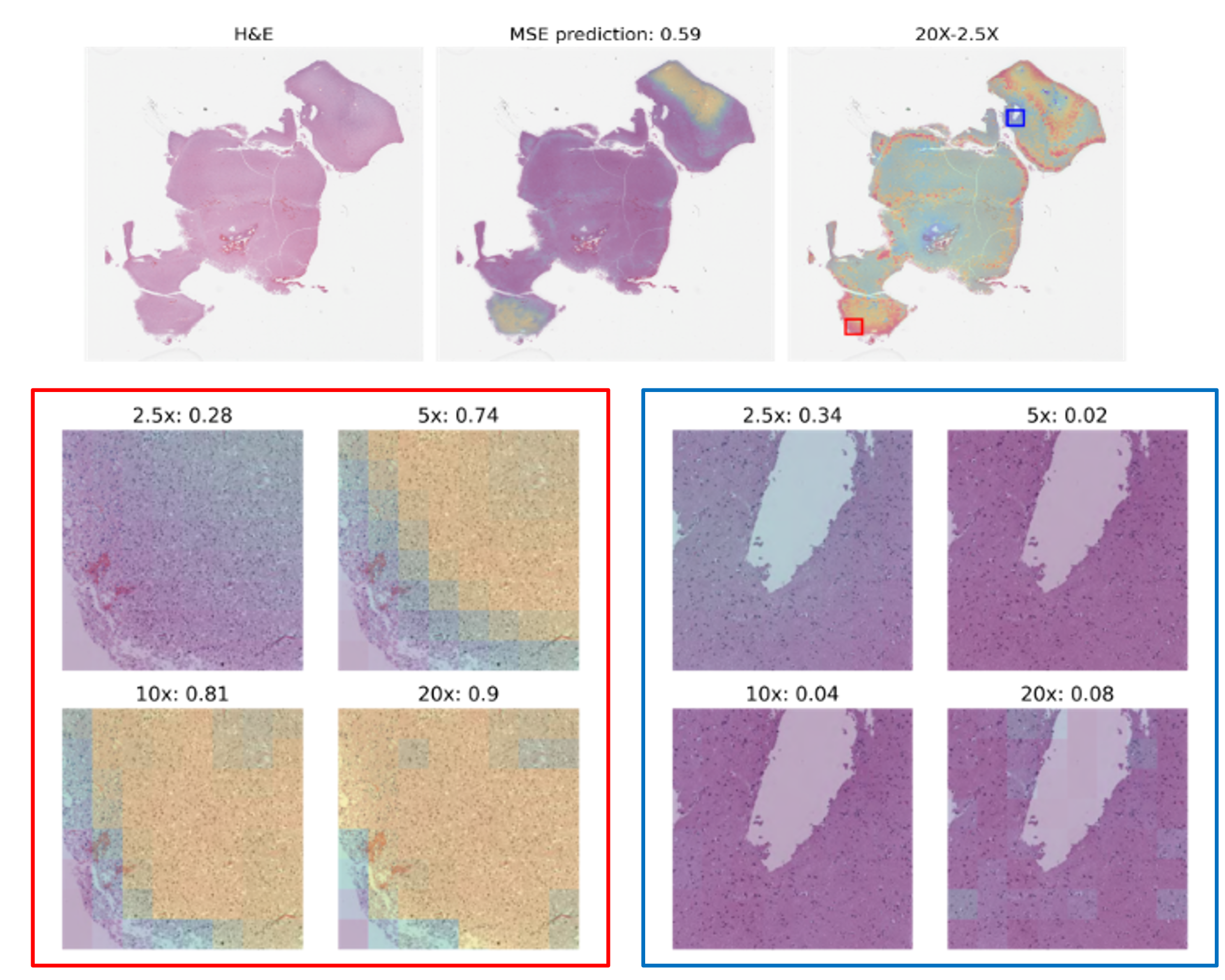


**Supplemental Figure 4:** Heatmap of the slide shown in Main Figure 4 A-I showing the difference in IDH prediction score at 20x and 2.5x (upper right panel), and details of pixel-level IDH prediction scores at areas with the greatest differential IDH prediction scores. The red-bounded box depicts an area with high IDH prediction at 20X and relatively lower IDH prediction at 2.5X. The blue bounded box depicts the converse. H&E = hematoxylin and eosin. MSE = Multiple Scale Ensemble. Software utilized the matlibplot 3.6.2 Python package available at https://matplotlib.org. Source code for sliding window software is available at https://github.com/Karenxzr/IDHmut/blob/main/Visualize.py.


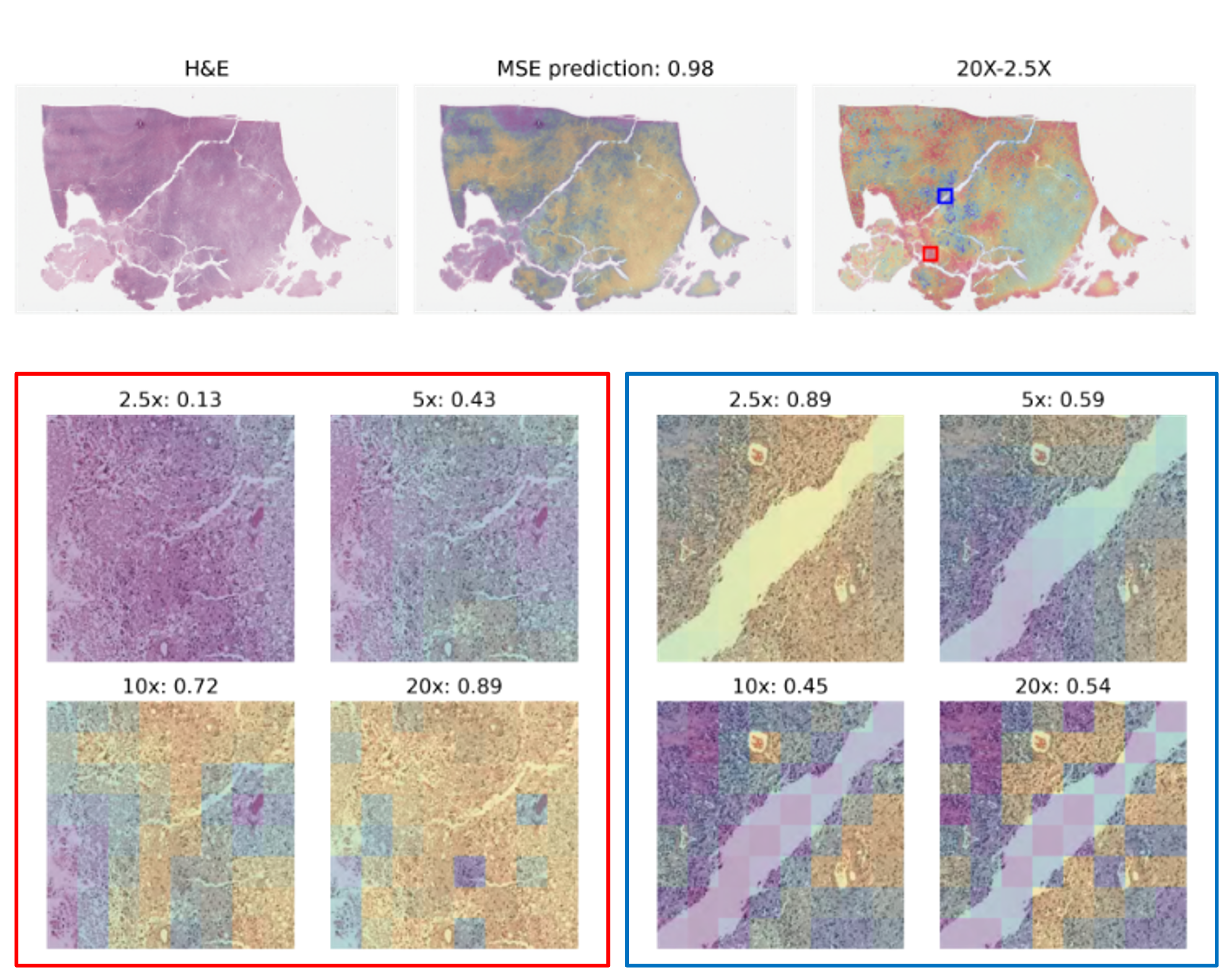


**Supplemental Figure 5:** Heatmap of the slide shown in Main Figure 4 A-I showing the difference in IDH prediction score at 20x and 2.5x (upper right panel), and details of pixel-level IDH prediction scores at areas with the greatest differential IDH prediction scores. The red-bounded box depicts an area with high IDH prediction at 20X and relatively lower IDH prediction at 2.5X. The blue bounded box depicts the converse. H&E = hematoxylin and eosin. MSE = Multiple Scale Ensemble. Software utilized the matlibplot 3.6.2 Python package available at https://matplotlib.org. Source code for sliding window software is available at https://github.com/Karenxzr/IDHmut/blob/main/Visualize.py.


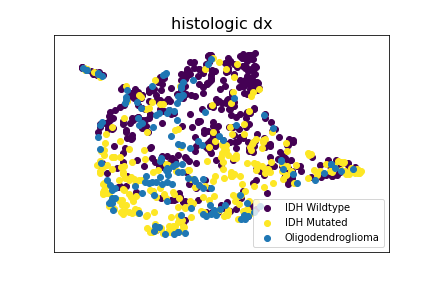


**Supplemental Figure 6:** Ground-truth histologic and molecular diagnoses of tiles extracted from the WCM test set from UMAP clustering shown in main figure 5.
