# Supplementary material for: Machine learning can aid in prediction of IDH mutation from H&E-stained histology slides in infiltrating gliomas
Source: Sci Rep. 2022 Dec 31;12:22623. doi: 10.1038/s41598-022-26170-6 (PMC9805452; doi:10.1038/s41598-022-26170-6)
Supplement: Supplementary file 2 — Supplementary Information 2. [file 41598_2022_26170_MOESM2_ESM.docx]

**Supplemental Tables:**

**Supplemental Table 1:**

|  |  |  |  |  |  |  |  |  |  |  |
| --- | --- | --- | --- | --- | --- | --- | --- | --- | --- | --- |
|  |  |  | **Slide-Level** | | | | **Patient-Level** | | | |
|  |  |  |  |  |  |  |  |  |  |  |
|  |  | **Accuracy** |  | **Sensitivity** | **Specificity** | **AUC** | **Accuracy** | **Sensitivity** | **Specificity** | **AUC** |
| **Pathologists** | **Pathologist 1** | **0.783 (0.781, 0.785)** |  | **0.714 (0.711, 0.717)** | **0.851 (0.849, 0.854)** | **0.873 (0.872, 0.875)** | **0.803 (0.801, 0.805)** | **0.731 (0.728, 0.734)** | **0.882 (0.879, 0.884)** | **0.896 (0.894, 0.898)** |
|  | **Pathologist 2** | **0.804 (0.802, 0.806)** |  | **0.815 (0.813, 0.818)** | **0.793 (0.791, 0.796)** | **0.881 (0.879, 0.882)** | **0.796 (0.794, 0.798)** | **0.797 (0.794, 0.800)** | **0.794 (0.791, 0.797)** | **0.901 (0.899, 0.903)** |
|  | **Two-pathologist Consensus** | **0.800 (0.798, 0.801)** |  | **0.760 (0.757, 0.762)** | **0.840 (0.837, 0.842)** | **0.906 (0.905, 0.907)** | **0.839 (0.837, 0.841)** | **0.799 (0.796, 0.802)** | **0.883 (0.88, 0.885)** | **0.920 (0.918, 0.921)** |
| **MSE** | **Multi-Scale Ensemble** | **0.771 (0.769, 0.773)** |  | **0.770 (0.767, 0.773)** | **0.772 (0.769, 0.774)** | **0.829 (0.827, 0.831)** | **0.809 (0.807, 0.811)** | **0.770 (0.767, 0.772)** | **0.853 (0.851, 0.856)** | **0.867 (0.866, 0.869)** |
| **Hybrid** | **MSE-Pathologist 1** | **0.794 (0.792, 0.796)** |  | **0.748 (0.745, 0.751)** | **0.840 (0.838, 0.842)** | **0.895 (0.894, 0.896)** | **0.824 (0.822, 0.826)** | **0.772 (0.769, 0.774)** | **0.883 (0.880, 0.885)** | **0.921 (0.920, 0.923)** |
|  | **MSE-Pathologist 2** | **0.822 (0.820, 0.824)** |  | **0.816 (0.814, 0.819)** | **0.829 (0.826, 0.831)** | **0.887 (0.885, 0.888)** | **0.832 (0.83, 0.834)** | **0.811 (0.808, 0.813)** | **0.855 (0.852, 0.858)** | **0.921 (0.919, 0.922)** |
|  | **MSE-Two-Pathologist Consensus** | **0.810 (0.809, 0.812)** |  | **0.793 (0.79, 0.795)** | **0.828 (0.826, 0.831)** | **0.897 (0.896, 0.899)** | **0.860 (0.858, 0.862)** | **0.825 (0.822, 0.827)** | **0.899 (0.897, 0.901)** | **0.928 (0.927, 0.929)** |
|  |  |  |  |  |  |  |  |  |  |  |

**Supplemental table 1:** Slide-level and patient-level performance on the WCM test dataset comparing individual pathologists, two-pathologist consensus, MSE model, and hybrid pathologist-MSE models.

**Supplemental Table 2:**

|  |  | | |  | | |  | | |  | |  | |  | |  | |  | |  | | |
| --- | --- | --- | --- | --- | --- | --- | --- | --- | --- | --- | --- | --- | --- | --- | --- | --- | --- | --- | --- | --- | --- | --- |
|  | | |  | | | Slide Level | | | | | | | | Patient Level | | | | | | | |  |
|  | | |  | | |  |  |  |  |  |  |  |  |  |  |  |  |  |  |  |  |  |
|  | | |  | | | Accuracy | | | Sensitivity | | Specificity | | AUC | | Accuracy | | Sensitivity | | Specificity | | AUC |  |
| 2.5X | | | Validation | | | 0.867  (0.864-0.870) | | | 0.934  (0.931-0.937) | | 0.800  (0.795-0.805) | | 0.95  (0.948-0.952) | | 0.795  (0.79-0.800) | | 0.845  (0.839-0.852) | | 0.754  (0.747-0.761) | | 0.909  (0.905-0.912) |  |
|  |  |  | Test | | | 0.882  (0.879-0.884) | | | 0.832  (0.828-0.836) | | 0.931  (0.928-0.934) | | 0.959  (0.958-0.961) | | 0.842  (0.838-0.846) | | 0.816  (0.81-0.821) | | 0.87  (0.865-0.876) | | 0.952  (0.949-0.954) |  |
|  |  |  | WCM Test | | | 0.707  (0.705-0.709) | | | 0.643  (0.64-0.646) | | 0.772  (0.769-0.774) | | 0.787  (0.785-0.789) | | 0.725  (0.723-0.728) | | 0.664  (0.661-0.667) | | 0.793  (0.79-0.796) | | 0.804  (0.802-0.807) |  |
| 5X | | | Validation | | | 0.848  (0.846-0.851) | | | 0.967  (0.965-0.969) | | 0.73  (0.725-0.735) | | 0.886  (0.883-0.889) | | 0.828  (0.823-0.832) | | 0.923  (0.918-0.928) | | 0.75  (0.743-0.756) | | 0.922  (0.919-0.925) |  |
|  |  |  | Test | | | 0.916  (0.913-0.918) | | | 0.933  (0.93-0.936) | | 0.898  (0.895-0.902) | | 0.989  (0.988-0.989) | | 0.873  (0.87-0.877) | | 0.876  (0.871-0.881) | | 0.87  (0.865-0.876) | | 0.984  (0.983-0.985) |  |
|  |  |  | WCM Test | | | 0.731  (0.729-0.733) | | | 0.839  (0.836-0.841) | | 0.623  (0.62-0.626) | | 0.807  (0.805-0.809) | | 0.773  (0.771-0.776) | | 0.851  (0.848-0.853) | | 0.688  (0.684-0.691) | | 0.852  (0.85-0.854) |  |
| 10X | | | Validation | | | 0.898  (0.896-0.901) | | | 1.000  (1.000-1.000) | | 0.797  (0.792-0.801) | | 0.941  (0.939-0.943) | | 0.896  (0.892-0.899) | | 1  (1-1) | | 0.81  (0.804-0.816) | | 0.981  (0.979-0.982) |  |
|  |  |  | Test | | | 0.966  (0.965-0.968) | | | 0.933  (0.930-0.936) | | 1  (1-1) | | 0.993  (0.993-0.994) | | 0.966  (0.965-0.968) | | 0.933  (0.93-0.936) | | 1  (1-1) | | 0.993  (0.993-0.994) |  |
|  |  |  | WCM Test | | | 0.782  (0.78-0.784) | | | 0.816  (0.813-0.818) | | 0.748  (0.745-0.751) | | 0.848  (0.847-0.850) | | 0.823  (0.822-0.825) | | 0.824  (0.821-0.827) | | 0.823  (0.82-0.826) | | 0.881  (0.88-0.883) |  |
| 20X | | | Validation | | | 0.932  (0.930-0.934) | | | 1.000  (1.000-1.000) | | 0.864  (0.86-0.868) | | 0.991  (0.99-0.991) | | 0.931  (0.929-0.934) | | 1  (1-1) | | 0.875  (0.87-0.881) | | 0.981  (0.98-0.982) |  |
|  |  |  | Test | | | 0.818  (0.815-0.821) | | | 0.867  (0.864-0.871) | | 0.768  (0.763-0.773) | | 0.942  (0.941-0.944) | | 0.839  (0.835-0.843) | | 0.812  (0.806-0.818) | | 0.868  (0.862-0.873) | | 0.967  (0.965-0.969) |  |
|  |  |  | WCM Test | | | 0.677  (0.675-0.679) | | | 0.526  (0.523-0.53) | | 0.828  (0.826-0.831) | | 0.781  (0.779-0.783) | | 0.726  (0.724-0.728) | | 0.582  (0.578-0.585) | | 0.885  (0.883-0.887) | | 0.837  (0.835-0.839) |  |
| Multiscale Ensemble | | | Validation | | | 0.882  (0.879-0.885) | | | 0.967  (0.965-0.969) | | 0.797  (0.792-0.801) | | 0.979  (0.978-0.98) | | 0.861  (0.857-0.865) | | 0.923  (0.918-0.928) | | 0.81  (0.804-0.816) | | 0.981  (0.979-0.982) |  |
|  |  |  | Test | | | 0.949  (0.948-0.951) | | | 0.933  (0.93-0.936) | | 0.966  (0.964-0.968) | | 0.988  (0.987-0.988) | | 0.936  (0.933-0.939) | | 0.876  (0.871-0.881) | | 1  (1-1) | | 0.984  (0.983-0.985) |  |
|  |  |  | WCM Test | | | 0.771  (0.769-0.773) | | | 0.77  (0.767-0.773) | | 0.772  (0.769-0.774) | | 0.829 (0.827-0.831) | | 0.809  (0.807-0.811) | | 0.77  (0.767-0.772) | | 0.853  (0.851-0.856) | | 0.867  (0.866-0.869) |  |
|  |  | | |  | | |  | | |  | |  | |  | |  | |  | |  | | |

**Supplemental table 2:** Model performances and 95% confidence intervals for the single-scale and multiscale ensembles, evaluated over the TCGA validation and test datasets, and the independent WCM test dataset.
